# Neopterin Is a Cerebrospinal Fluid Marker for Treatment Outcome Evaluation in Patients Affected by *Trypanosoma brucei gambiense* Sleeping Sickness

**DOI:** 10.1371/journal.pntd.0002088

**Published:** 2013-02-28

**Authors:** Natalia Tiberti, Veerle Lejon, Alexandre Hainard, Bertrand Courtioux, Xavier Robin, Natacha Turck, Krister Kristensson, Enock Matovu, John Charles Enyaru, Dieudonné Mumba Ngoyi, Sanjeev Krishna, Sylvie Bisser, Joseph Mathu Ndung′u, Philippe Büscher, Jean-Charles Sanchez

**Affiliations:** 1 Translational Biomarker Group, Department of Human Protein Sciences, University of Geneva, Geneva, Switzerland; 2 Department of Biomedical Sciences, Institute of Tropical Medicine, Antwerp, Belgium; 3 Institut National de la Santé et de la Recherche Médicale (INSERM), UMR1094, Tropical Neuroepidemiology, Limoges, France; 4 Institute of Neuroepidemiology and Tropical Neurology, School of Medicine, CNRS FR 3503 GEIST, University of Limoges, Limoges, France; 5 Department of Neuroscience, Karolinska Institutet, Stockholm, Sweden; 6 Department of Veterinary Parasitology and Microbiology, School of Veterinary Medicine, Makerere University, Kampala, Uganda; 7 Department of Biochemistry, College of Natural Sciences, Makerere University, Kampala, Uganda; 8 Department of Parasitology, Institut National de Recherche Biomédicale, Kinshasa, D. R. Congo; 9 Centre for Infection, Division of Cellular and Molecular Medicine, St. George's, University of London, London, United Kingdom; 10 Foundation for Innovative New Diagnostics (FIND), Geneva, Switzerland; Hospital Universitário, Brazil

## Abstract

**Background:**

Post-therapeutic follow-up is essential to confirm cure and to detect early treatment failures in patients affected by sleeping sickness (HAT). Current methods, based on finding of parasites in blood and cerebrospinal fluid (CSF) and counting of white blood cells (WBC) in CSF, are imperfect. New markers for treatment outcome evaluation are needed. We hypothesized that alternative CSF markers, able to diagnose the meningo-encephalitic stage of the disease, could also be useful for the evaluation of treatment outcome.

**Methodology/Principal findings:**

Cerebrospinal fluid from patients affected by *Trypanosoma brucei gambiense* HAT and followed for two years after treatment was investigated. The population comprised stage 2 (S2) patients either cured or experiencing treatment failure during the follow-up. IgM, neopterin, B2MG, MMP-9, ICAM-1, VCAM-1, CXCL10 and CXCL13 were first screened on a small number of HAT patients (n = 97). Neopterin and CXCL13 showed the highest accuracy in discriminating between S2 cured and S2 relapsed patients (AUC 99% and 94%, respectively). When verified on a larger cohort (n = 242), neopterin resulted to be the most efficient predictor of outcome. High levels of this molecule before treatment were already associated with an increased risk of treatment failure. At six months after treatment, neopterin discriminated between cured and relapsed S2 patients with 87% specificity and 92% sensitivity, showing a higher accuracy than white blood cell numbers.

**Conclusions/Significance:**

In the present study, neopterin was highlighted as a useful marker for the evaluation of the post-therapeutic outcome in patients suffering from sleeping sickness. Detectable levels of this marker in the CSF have the potential to shorten the follow-up for HAT patients to six months after the end of the treatment.

## Author Summary

The reduction of the number of lumbar punctures performed during the follow-up of patients affected by sleeping sickness (HAT) is considered a research priority. Follow-up, consisting of the examination of cerebrospinal fluid (CSF) for presence of parasites and for the number of leukocytes, is necessary to assess treatment outcome. However, diagnosis of treatment failure is still imperfect and WHO encourages improvements in defining criteria. Many studies have attempted to standardize actual methods and to define a cut-off for the number of white blood cells in CSF to define relapses, while only few have proposed alternatives to current practice. Here we show that neopterin, already proven to be a powerful marker for staging *T. b. gambiense* HAT, is also useful in evaluating post-therapeutic outcome. The measurement of neopterin concentration in CSF during the follow-up may allow reduction in the number of lumbar punctures from five to three for the majority of cured patients.

## Introduction

Sleeping sickness, also known as human African trypanosomiasis (HAT), is a neglected parasitic disease widespread in sub-Saharan Africa where it mainly afflicts rural communities [1]. According to the most recent published data, 10'000 new cases were reported in 2009 [2]. More than ninety percent of HAT cases are caused by *Trypanosoma brucei gambiense* parasite, which is responsible for a chronic disease in Western and Central Africa [2]. Without treatment the disease progresses through two stages. Immediately after infection the proliferation of the parasites in blood and lymph, gives rise to the haemolymphatic first stage (stage 1, S1). If stage 1 patients are not treated, the disease progresses to the second meningo-encephalitic stage (stage 2, S2) as a consequence of the penetration of the parasites into the central nervous system (CNS) [2]. HAT patients need to be treated according to their stage. Thus, S1 patients should receive pentamidine treatment, while S2 patients can be treated using melarsoprol, eflornithine or NECT (nifurtimox-eflornithine combination therapy) [3], [4]. However, patients cannot be considered cured immediately after treatment as parasites may persist in the host and the disease may reappear later on [5] as a consequence of treatment failure. To assess the efficacy of the treatment, patients need to be followed for two years to detect relapses, or to confirm recovery [6]. WHO still recommends 5 follow-up visits performed at the end of the treatment (EoT) and at 6, 12, 18 and 24 months after treatment [6]. Visits consist of clinical assessments, examination of blood and cerebrospinal fluid (CSF) for the presence of trypanosomes and evaluation of the number of white blood cells (WBC) in the CSF. Optimal criteria to detect relapses accurately and early, when trypanosomes are not yet detectable, are being investigated. Several studies have tried to determine a cut-off for the number of WBCs, to predict relapses and to shorten the follow-up to less than 24 months after treatment [5], [7], [8]. Recently, an algorithm based on the CSF WBC count at 6 and 12 months has been proposed and showed a high potential in shortening patient follow-up as soon as 6 months after treatment [5], [8]. However, the counting of WBC still has weaknesses, such as limited specificity and reproducibility, as already highlighted for the staging of HAT patients [9]. New surrogate markers to assess the post-therapeutic outcome therefore represent an unmet need, as highlighted by WHO [10], [11].

Very few alternative markers in CSF have been evaluated so far. These include DNA detection by PCR [12], [13], IgM and trypanosome specific antibodies, total proteins and the level of the anti-inflammatory cytokine IL-10 [14]. However, when assessed at 6 and 12 months after treatment, they showed lower accuracy as outcome predictors compared to WBC.

We hypothesize that newly described CSF staging markers [15]–[23], able to indicate the presence of the second stage of sleeping sickness, could also indicate a reappearance of the infection in S2 patients after treatment. IgM, B2MG, CXCL13, CXCL10, MMP-9, VCAM-1, ICAM-1 and neopterin were first tested on a small cohort of HAT patients followed after treatment to carry out a preliminary selection of molecules with highest accuracy as outcome predictors. Markers with the highest accuracy (neopterin and CXCL13) were further validated on a larger number of patients and compared to WBC to assess the treatment outcome.

## Methods

### Ethics statement

The THARSAT study, from which patients originated, was approved by the Ministry of Health of the Democratic Republic of the Congo and by the Commission for Medical Ethics of the Institute of Tropical Medicine Antwerp, Belgium (reference 04441472). All patients, or their legal representatives, gave written informed consent before enrolment. All patients had the possibility to withdraw from the study at any moment.

### Patients

The present study was designed into two parts: a first screening of 8 markers on a small population, followed by the verification of the two most promising markers on a larger cohort.

All patients were enrolled by either active or passive case finding in the Democratic Republic of the Congo as part of the THARSAT study [8]. Inclusion and exclusion criteria are reported elsewhere [8].

All patients had parasitologically confirmed HAT, either as primary cases (no previous HAT treatment) or as secondary cases (previously treated for HAT). Stage determination was performed through CSF examination for number of leukocytes and presence of parasites following modified single centrifugation [24]. Stage was defined according to WHO guidelines, i.e. stage 1 when WBC ≤5 cells/µL and absence of parasites, stage 2 when WBC>5 cells/µL and/or parasites detected in CSF [6]. Patients were treated according to their stage as reported by Mumba Ngoyi *et al*. [8]. After treatment, patients were followed up with visits planned at the end of the treatment (EoT) and at 3, 6, 12, 18 and 24 months after treatment. Blood and CSF examinations were performed at each follow-up visit and outcome was determined as recommended by WHO [8], [10]. Briefly, cured patients were defined based on absence of trypanosomes during the follow-up and CSF WBC ≤20/µL at 24 months for stage 2, or CSF WBC ≤5/µL for stage 1. Confirmed relapses were diagnosed following the finding of parasites in CSF at any follow-up visit. Probable relapses were diagnosed following an increased count of WBC (more than 30 WBC/µL compared to the lowest number of WBC obtained during the previous FU examinations) and/or aggravation of neurological signs, or WBC>20/µL at 24 months [8]. Patients classified either as confirmed or probable relapses were considered as treatment failures.

The patients investigated in the present study were selected among the 360 participants of the THARSAT study [8], after exclusion of those who died prior or during the follow-up, relapses of early stage patients, patients lost during the follow-up or for whom 2 or more interim follow-up visits were missing. Furthermore, patients whose diagnosis of relapse was based on the finding of parasites in blood (n = 8) were also excluded, as they could potentially represent re-infection cases.

The screening cohort comprised S1 (n = 19) and S2 (n = 78) primary cases. All S2 patients included in the screening cohort received melarsoprol treatment and only cases of treatment failure (i.e. S2 relapse) defined as confirmed relapse were chosen. Selected cured and relapsed S2 patients were matched for age and sex. Characteristics at baseline, i.e. observed at the moment of the diagnosis and before the treatment, of patients included in the screening cohort are reported in Supporting Table S1.

The verification cohort (n = 242) comprised all patients of the THARSAT study considered eligible for the present study and for whom enough CSF sample volume was available to perform all the analyses. Eighty six patients were included in both screening and verification cohort. The characteristics at baseline of the verification cohort are reported in Table 1. More details on the verification cohort are reported in Supporting Figure S1.

**Table 1:** Characteristics at baseline of the verification cohort.

	S1 cured (n = 21)	S2 cured (n = 114)	S2 relapsed* (n = 107)
**Demography**			
Sex, F (n)†	14 (66.7%)	28 (24.6%)	33 (35.3%)
Age, years [mean ± SD]‡	37.3 [±13]	34 [±12.4]	34.3 [±12.4]
**Pre-treatment CSF examination**			
Trypanosome positive, n	0	104	100
WBC/µL (median, range)	3 [1]–[5]	213 [2–1940]	267 [8–2064]
**Neurological signs**			
Absent	13	15	13
Present	8	99	94
**Treatment**			
P	21	0	0
E	0	42	2
M	0	40	99
M-E	0	1	0
M-N	0	31	6
Secondary case, n∥	0	66	9

* n = 27, probable relapse.

† No significant difference between S2 cured and S2 relapsed, Fisher's exact test.

‡ No significant difference between S2 cured and S2 relapsed, Mann-Whitney *U* test.

∥ Secondary case: patients already treated once for HAT.

P: pentamidine treatment; E: eflornithine treatment; M: melarsoprol treatment; M-E: combination of melarsoprol and eflornithine; M-N: combination of melarsoprol and nifurtimox. More details on treatment regimens are reported in [8].

### Immunoassays

Cerebrospinal fluid levels of neopterin (Brahms, Thermo Fisher Scientific, Germany), IgM (ICL, OR, USA), B2MG (Calbiotech, CA, USA) and CXCL13 (R&D Systems, UK and RayBiotech, GA, USA), were measured using commercially available ELISA assays. The levels in CSF of CXCL10, MMP-9, ICAM-1 and VCAM-1 were measured using multiplex bead suspension assays (R&D Systems, UK).

All assays were performed according to manufacturer's instructions and the inter-assay variability was evaluated using quality controls (coefficient of variation - CV<20%). A limit of detection (LOD, corresponding to the mean measured concentration for the lowest standard less 2 standard deviations) was calculated for each assay. To all outliers (≤LOD) a value corresponding to the mean of LODs divided by 2 was assigned.

### Statistical analysis

All statistical analyses were performed using IBM SPSS Statistics version 20.0.0 (IBM, NY, USA) and STATA version 11.0 (StataCorp LP, TX, USA). Receiver operating characteristic (ROC) curves, area under the ROC curve (AUC), corrected partial AUC (pAUC), sensitivity (SE) and specificity (SP) were computed using the pROC package for S+ version 8.1 (TIBCO, Software Inc.).

All statistical tests were two tailed and significance level was set at 0.05. Comparison between two groups was performed with the Mann-Whitney *U* test for independent variables or using the Wilcoxon signed rank test for dependent variables. The accuracy of the markers in discriminating between cure and relapse was evaluated considering only stage 2 patients.

#### First analysis – screening

ROC analysis was performed to assess the accuracy of the markers in discriminating between cured and relapsing patients. The AUC and the cut-off corresponding to the highest combination of specificity and sensitivity were calculated. Comparison was done between the levels of the markers measured in S2 patients at the moment of the relapse taking cured S2 patients at matched time points. The two best markers were selected for further analyses.

#### Second analysis – verification

Baseline risk factors, i.e. pre-treatment, for relapse were assessed using logistic regression and calculating the relative risk, prior and after adjustment for treatment. The following baseline variables were investigated for their association with an increased risk of treatment failure: sex, age, treatment, secondary cases, incomplete treatment, presence of parasites in CSF, number of WBC in CSF, presence of neurological signs and CSF concentrations of neopterin and CXCL13. The threshold in concentration of the two markers was determined using ROC curves and comparing their baseline levels in the two categories of late stage patients (cured and relapsed).

The accuracy for the prediction of treatment outcome was evaluated for neopterin, CXCL13 and WBC at three, six and twelve months after treatment. Eighteen and 24 months were not tested due to the low number of patients experiencing a relapse later than 12 months after treatment in the THARSAT study. For each marker and at each time point of the follow-up a highly sensitive cut-off was selected within an area under the ROC curve comprised between 90 and 100% sensitivity (partial AUC - pAUC) [25]. Positive and negative likelihood ratios (LR+ and LR−, respectively) were calculated to better evaluate the association of the levels of the markers and the final outcome at the 3 time points of follow-up considered [26].

## Results

### First analysis - screening

The first analysis consisted in the evaluation of IgM, B2MG, CXCL10, CXCL13, MMP-9, VCAM-1, ICAM-1 and neopterin on a cohort of 97 patients.

According to the AUC, neopterin and CXCL13 showed the highest accuracy in discriminating S2 cured and S2 relapsed patients (Table 2). Neopterin showed a higher AUC, and both neopterin and CXCL13 showed higher sensitivity than the counting of leukocytes.

**Table 2:** Performance of the markers on the screening cohort.

Marker	AUC% (95%CI)	Cut-off	SP% (95%CI)	SE% (95%CI)
Neopterin [nmol/L]	99.1 (97.7–100)	64.4	100 (100–100)	92.3 (82.1–100)
CXCL13 [pg/mL]	94.0 (88.9–99.2)	125.5	84.6 (71.8–94.9)	92.3 (82.1–100)
ICAM-1 [ng/mL]	93.2 (87.0–99.4)	2.1	92.3 (82.1–100)	87.2 (76.9–97.4)
CXCL10 [pg/mL]	90.0 (83.6–98.2)	2308.4	84.6 (71.8–94.9)	89.7 (79.5–97.4)
B2MG [ng/mL]	89.2 (81.4–97.1)	2486.5	94.9 (87.2–100)	76.9 (64.1–89.7)
MMP-9 [pg/mL]	88.8 (80.8–96.9)	814.8	84.6 (71.8–94.9)	87.2 (76.9–97.4)
VCAM-1 [ng/mL]	86.2 (77.4–95)	38.5	94.9 (87.2–100)	69.2 (53.9–82.1)
IgM [µg/mL]	81.2 (71.6–90.8)	75.9	66.7 (51.3–79.5)	87.2 (76.9–97.4)
WBC (Cells/µL)	96.9 (93.9–100)	44.5	94.9 (87.2–100)	89.7 (79.5–97.4)

Number of patients: 39 S2 relapsed vs. 39 S2 cured.

SP%: specificity %; SE%: sensitivity%; 95%CI: 95% confidence interval.

The reported cut-off corresponds to the best combination of specificity and sensitivity obtained for each marker.

The ability of neopterin and CXCL13 in following the disease progression in the different categories of patients was further confirmed through the kinetic profiles, where an increased concentration of the two markers in relapsing patients was highlighted (Supporting Figure S2).

### Second analysis - verification

#### Pre-treatment evaluation of the markers

Neopterin and CXCL13 were investigated on a larger population comprising 242 patients (Table 1, Supporting Figure S1). Baseline characteristics of S2 patients, cured and relapsed, were first analysed to evaluate whether they could already represent a risk of treatment failure (Table 3). An increased risk of relapse was associated with the received treatment and with the condition of being a primary case, i.e. never treated before for HAT. As these two conditions are strictly associated (primary cases were preferentially treated with melarsoprol), new relative risks were calculated after adjustment for treatment. This analysis highlighted an increased risk of relapse for patients presenting parasites in the CSF and a high count of WBC (≥100/µl) at diagnosis.

**Table 3:** Baseline risk factors for treatment failure in late stage patients.

	*Treatment failure*	*Unadjusted*		*Adjusted for TT*	
Baseline variable	n/N	RR	p value	RR	p value
Sex					
F	33/61	1		1	
M	74/160	0.85	0.28	0.88	0.31
Age					
<25	27/55	1		1	
25–39	43/94	0.93	0.69	1	0.99
>40	37/72	1.05	0.80	0.91	0.54
Treatment					
E	2/44	1			
M	99/139	15.67	<0.0001		
M-E	0/1	0.00	<0.0001		
M-N	6/37	3.57	0.11		
Secondary case					
No	98/144	1		1	
Yes	9/77	0.17	<0.0001	0.94	0.90
Incomplete treatment					
No	92/197	1		1	
Yes	15/24	1.34	0.10	1	0.96
Parasites in CSF					
T−	7/17	1		1	
T+	100/204	1.19	0.56	1.83	0.04
CSF WBC/µL					
<100	17/46	1		1	
100–199	26/50	1.40	0.15	1.64	0.02
200–399	32/69	1.25	0.39	1.52	0.04
≥400	32/56	1.54	0.05	1.32	0.18
Neurological signs					
NS−	12/27	1		1	
NS+	95/194	1.1	0.67	1.28	0.24
Neopterin [nmol/L]					
≤261.6	35/96	1		1	
>261.6	72/125	1.58	0.003	1.75	<0.0001
CXCL13 [pg/mL]					
≤1734.1	17/50	1		1	
>1734.1	90/171	1.55	0.04	1.55	0.02

TT: treatment; E: eflornithine treatment; M: melarsoprol treatment; M-E: combination of melarsoprol and eflornithine; M-N: combination of melarsoprol and nifurtimox.

n/N: n = number of patients experiencing treatment failure, N = total number of patients.

RR: risk ratio.

T−: absence of parasite in CSF; T+: presence of parasites in CSF.

NS−: absence of neurological sings, NS+: presence of neurological sings.

Age and WBC/µL categories were defined according to Mumba Ngoyi D. *et al.* [8].

At the time of the diagnosis, i.e. before treatment, the levels of neopterin and CXCL13 were higher in S2 patients, cured or relapsed, compared to S1 (p value<0.0001, Kruskal-Wallis test, followed by post-test paired comparison). Only neopterin was able to significantly discriminate (p<0.05) between cured S2 patients and relapsed S2 patients already at baseline with AUC of 60% (52.8–67.74 95% CI), while CXCL13 and WBC showed AUC of 55% (49.2–61, 95%CI) and 56.8% (49.3–64.4), respectively. However, no significant differences were observed between the ROC curves (DeLong's test for two correlated ROC curves) (data not shown).

When the best cut-offs for discrimination between S2 cured and relapsed patients at baseline (neopterin 261.6 nmol/L and CXCL13 1734.1 pg/mL) were used to dichotomize the population, patients presenting high baseline levels of the markers were associated to a significantly higher risk for treatment failure (Table 3).

#### Evolution of the markers after treatment

A significant decrease in CSF concentration of neopterin, CXCL13 and white blood cells was observed in all S2 patients after treatment (p value<0.0001, Wilcoxon signed rank test for paired samples) (data not shown).

**Figure 1:**
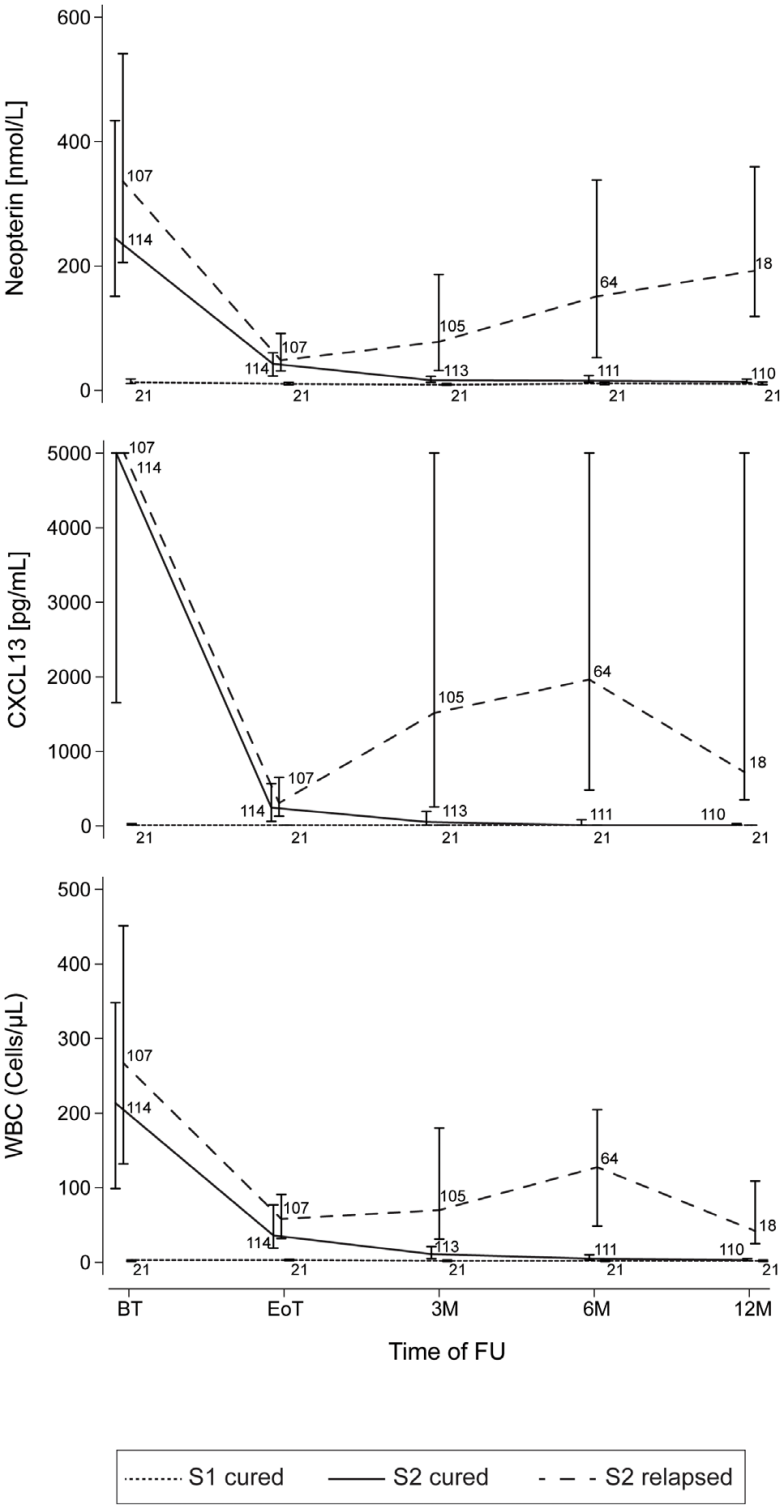
Kinetics of neopterin, CXCL13 and WBC during the follow-up. The variation in concentrations of the three markers in S1 cured patients, S2 cured patients and S2 relapsing patients are represented. Median concentrations at each time point are reported. Bars represent inter-quartile intervals. Numbers on the graphs represent the number of CSF samples assessed at each time point for each category of HAT patients. BT: before treatment; EoT: end of treatment; 3 M, 6 M, 12 M: 3, 6, 12 months after treatment. FU: follow-up.

The evolution of the two markers and of WBC during further follow-up of HAT patients is represented in Figure 1. The levels of neopterin, CXCL13 and of WBC in S1 cured patients remained constantly lower than in S2 patients. In late stage patients confirmed to be cured at the end of the follow-up neopterin, CXCL13 and WBC reached levels comparable to those observed in S1 patients cured already 3 months after treatment (Figure 1).

In those patients experiencing a relapse, the levels of the two markers and of WBC, after having decreased following treatment administration, started to increase at 3 months after treatment when 43 patients out of 105 already had a relapse or a probable relapse. Similarly, higher median concentration of neopterin and CXCL13 were observed in relapsing patients 6 and 12 months after treatment compared to cured S2 patients.

ROC curves were built to assess the performance of neopterin, CXCL13 and WBC count in discriminating between cured S2 patients and S2 patients having a relapse 3, 6 and 12 months after treatment. Partial AUC (pAUC) between 90 and 100% of sensitivity and the best cut-off within this area were calculated (Table 4). When measured in the CSF taken 3 months after treatment, neopterin and CXCL13 showed accuracy comparable to the one of WBC. However, six months after treatment, neopterin was a better predictor of the outcome than both CXCL13 and WBC with a negative likelihood ratio <0.1 indicating a strong correlation between the levels of this marker and the ruling out of cured patients. At this time point, neopterin, at a CSF concentration of 28 nmol/L was able to correctly classify as cured 97 out of 111 patients, with only 5 false negatives. Similarly, a WBC count of <11 cells/µL could correctly predict 90 out of 111 cured patients with 6 false negatives, while CXCL13 could only predict 71 out of 111 cured patients.

**Table 4:** Performances of neopterin, CXCL13 and WBC in discriminating between S2 cured and S2 relapsed patient.

Marker, Tx	AUC%	pAUC%	Cut-Off	SP% (95%CI)	SE% (95%CI)	LR+	LR−	TN	FN
**Neopterin [nmol/L]**									
3 M	90	69.3	17.3	57.5 (48.7–66.4)	93.3 (88.6–98.1)	2.2	0.12	65	7
6 M	93.9	75.8	27.9	87.4 (81.1–92.8)	92.2 (84.4–98.4)	7.3	0.09	97	5
12 M	98.4	93.3	41.4	97.3 (93.6–100)	94.4 (83.3–100)	35.0	0.06	107	1
**CXCL13 [pg/mL]**									
3 M	86	60.4	58.9	52.2 (42.5–61.1)	90.5 (84.8–96.2)	1.9	0.18	59	10
6 M	92.4	69.7	53.2	64 (55–73)	90.6 (82.8–96.9)	2.5	0.15	71	6
12 M	94.7	78.3	76.7	87.3 (80.9–92.7)	94.4 (83.3–100)	7.4	0.06	96	1
**WBC (Cells/µL)**									
3 M	87.1	64.5	14.5	62.8 (54–71.7)	90.5 (84.8–96.2)	2.4	0.15	71	10
6 M	93.3	71.4	11.5	81.1 (73.9–88.3)	90.6 (82.8–96.9)	4.8	0.12	90	6
12 M	94.4	70.9	9.5	92.7 (87.3–97.3)	94.4 (83.3–100)	12.9	0.06	102	1

3 M, 6 M, 12 M: 3, 6, 12 months after treatment.

Number of patients: 3 M cured n = 113, relapsed m = 105; 6 M cured n = 111, relapsed n = 64; 12 M cured n = 110, relapsed n = 18.

*pAUC was calculated between 90 and 100% of sensitivity.

SP% = specificity%; SE% = sensitivity%.

Cut-off corresponds to the best combination of SP and SE within the pAUC.

LR+: positive likelihood ratio, LR−: negative likelihood ratio.

TN: true negatives, number of cured patients correctly classified.

FN: false negatives, number of relapsing patients wrongly classified as cured.

Finally, twelve months after treatment neopterin showed strong power to rule in (LR+ 35) and to rule out (LR− 0.06) S2 patients. At a concentration of 41.4 nmol/L, it could correctly predict cure in 97% (93.6–100 95%CI) of cured patients with only 1 relapsing patient miss-classified, compared to the 93% predicted by WBC (87.3–97 95%CI). CXCL13 showed again a lower accuracy, being able to predict cure in 87% of cured patients (80.9–92.7% CI) (Table 4).

## Discussion

The long post-therapeutic follow-up for patients affected with sleeping sickness is a major limitation in the management of HAT patients [6]. A gold standard to detect treatment failures is still missing [10]. New tools to achieve a better evaluation of treatment outcome, in terms of early detection of relapses and reduction of the time of follow-up are absolutely needed [11]. The reduction of the current follow-up of two-years would not only have the advantage of reducing the number of lumbar punctures, but would also potentially increase the compliance rate, as after 6 months a decrease of the attendance rate has been reported [27].

In the present study, we investigated the ability of a number of molecules associated with an advanced stage of disease at diagnosis, as markers for treatment outcome [15].

The evaluation of IgM, B2MG, CXCL10, CXCL13, MMP-9, ICAM-1, VCAM-1 and neopterin was performed on a first cohort of patients (n = 97) followed after treatment. Neopterin, already highlighted as a powerful marker to stage *T. b. gambiense* HAT [15], resulted here to be the most accurate discriminator between cured and relapsed patients, together with the chemoattractant chemokine CXCL13.

The association of these molecules with the advanced stage of HAT at diagnosis, already reported [15], [19], was confirmed. Furthermore, their high CSF concentration at baseline showed a strong association with an increased risk of treatment failure. When assessed during the complete follow-up, both neopterin and CXCL13 were able to indicate the recurrence of brain disease, as their concentration significantly increased in association with relapse.

From a functional point of view, both markers might be associated with the immune-pathogenesis of HAT. CXCL13 is a chemokine involved in the recruitment of B and T lymphocytes to the site of inflammation and its potential involvement in HAT progression has already been proposed [19]. Neopterin is a catabolic product of the GTP [28] known as an indicator of the immune response activation, a central process in HAT late stage pathogenesis [29]. However, further investigations are needed to better understand the role of these molecules in both disease progression and reappearance.

Neopterin was here shown to be the most accurate marker for treatment outcome evaluation. When measured in the CSF of HAT patients 6 months after the end of the treatment, it was able to shorten the follow-up in 97 out of 111 S2 cured patients, thus potentially reducing the follow-up period and the number of lumbar punctures.

The present study has a number of limitations. In the verification population, treatment failures were diagnosed either based on the reappearance of the parasites in the CSF, or based on a high number of white blood cell count (probable relapses). This could represent a bias as white blood cells are not considered as a gold standard for the detection of relapses, but for some patients it was considered as a diagnostic criterion to which our markers were compared. Interestingly, the concentration of neopterin and the number of WBC, but not CXCL13, was significantly lower in suspected relapses compared to confirmed relapses 6 months after treatment (data not shown), suggesting that some physiopathological differences may characterize the two groups.

Most relapse cases included in the present study had received melarsoprol treatment, which was, at the moment of the study, the first line treatment in the Democratic Republic of the Congo. However, due to the high relapsing rate observed after melarsoprol treatment, the first treatment of choice for *T. b. gambiense* HAT has now changed to eflornithine and NECT therapies [3], [4]. Deeper investigations on a multi-centric cohort including recoveries and failures after treatments other than melarsoprol are needed, as already done for the counting of leukocytes [5], even if low failure rates with NECT have been reported so far [30].

Another drawback may rely in the lack of specificity of neopterin. This metabolite has been reported to be an indicator of the immune activation in other pathological conditions including HIV [31], [32]. However, we observed significantly lower levels of neopterin in patients affected by cerebral malaria or meningitis when compared to HAT patients (data not shown). Better insights on the role of neopterin in the physiopathology of disease relapses could be achieved through the use of animal models, as already done for IL-10 [33].

Due to its high ability in stage determination [15], developments for the translation of neopterin in an ASSURED test [34] for staging are ongoing. Here we extended the potential utility of this marker by showing its power as outcome predictor. In the present study, different cut-offs in neopterin concentration have been calculated, corresponding to the best performance of the marker at each time point of the follow-up. However, to be translated into clinical practice, a unique and highly accurate cut-off for the interim-follow up visits as well as a cut-off for the TOC visit should be determined, as it has been recently done for the WBC [5], [8].

The reduction of the follow-up from 24 months to a maximum of 12 months would have the major advantage of a decreased number of lumbar punctures for patients and, as a consequence, an increased attendance rate. However, a further improvement in patients' management would be the finding of test-of-cure markers in plasma. All molecules investigated here in CSF, were also assessed in the plasma of a small number of patients, but none of them was able to indicate the reappearance of the disease (data not shown).

In conclusion, the present study demonstrated the accuracy of neopterin in predicting and detecting treatment outcome for HAT patients. Due to its power as both staging [15] and follow-up marker for *T. b. gambiense* sleeping sickness, it is a promising candidate for further investigations in the field.

## Supporting Information

Figure S1 **Flow-chart describing the patients investigated for the verification analysis.** BT: before treatment; EoT: end of treatment; DE: disease evolution; 3 M, 6 M, 12 M, 18 M, 24 M: 3, 6, 12, 18, 24 months after treatment; ToC: test-of-cure. * Patients were included according to the criteria described by Mumba Ngoyi *et al.* [8]. ‡ Patients excluded from the present study: n = 8, presence of parasites in blood at the time of relapse; n = 4, stage 1 relapse; n = 37, death during the follow-up; n = 46, lost during the follow-up or missing more than one time of interim follow-up; n = 23, insufficient CSF sample. § One patient was considered cured at the end of the follow-up even if the last visit was done 18 months after treatment. Nomenclature assigned according to WHO/CDS/NTD/IDM/2007.1 [10].
(TIF)

Figure S2 **Kinetics of the eight molecules and of WBC assessed on the screening cohort.** The variation in concentrations of neopterin, CXCL13, CXCL10, ICAM-1, VCAM-1, B2MG, MMP-9, VCAM-1 and IgM, as well as the number of WBC in the CSF of S1 cured patients, S2 cured patients and S2 relapsing patients are represented. Median concentrations at each time point are reported. Bars represent inter-quartile intervals. Numbers on the graphs represent the number of CSF samples assessed at each time point for each category of HAT patients. BT: before treatment; EoT: end of treatment; 3 M, 6 M, 12 M: 3, 6, 12 months after treatment. FU: follow-up.
(TIF)

Table S1 Characteristics at baseline of the screening cohort.
(DOC)
