# Supplementary figures and images for: Neopterin Is a Cerebrospinal Fluid Marker for Treatment Outcome Evaluation in Patients Affected by Trypanosoma brucei gambiense Sleeping Sickness
Source: PLoS Negl Trop Dis. 2013 Feb 28;7(2):e2088. doi: 10.1371/journal.pntd.0002088 (PMC3585011; doi:10.1371/journal.pntd.0002088)

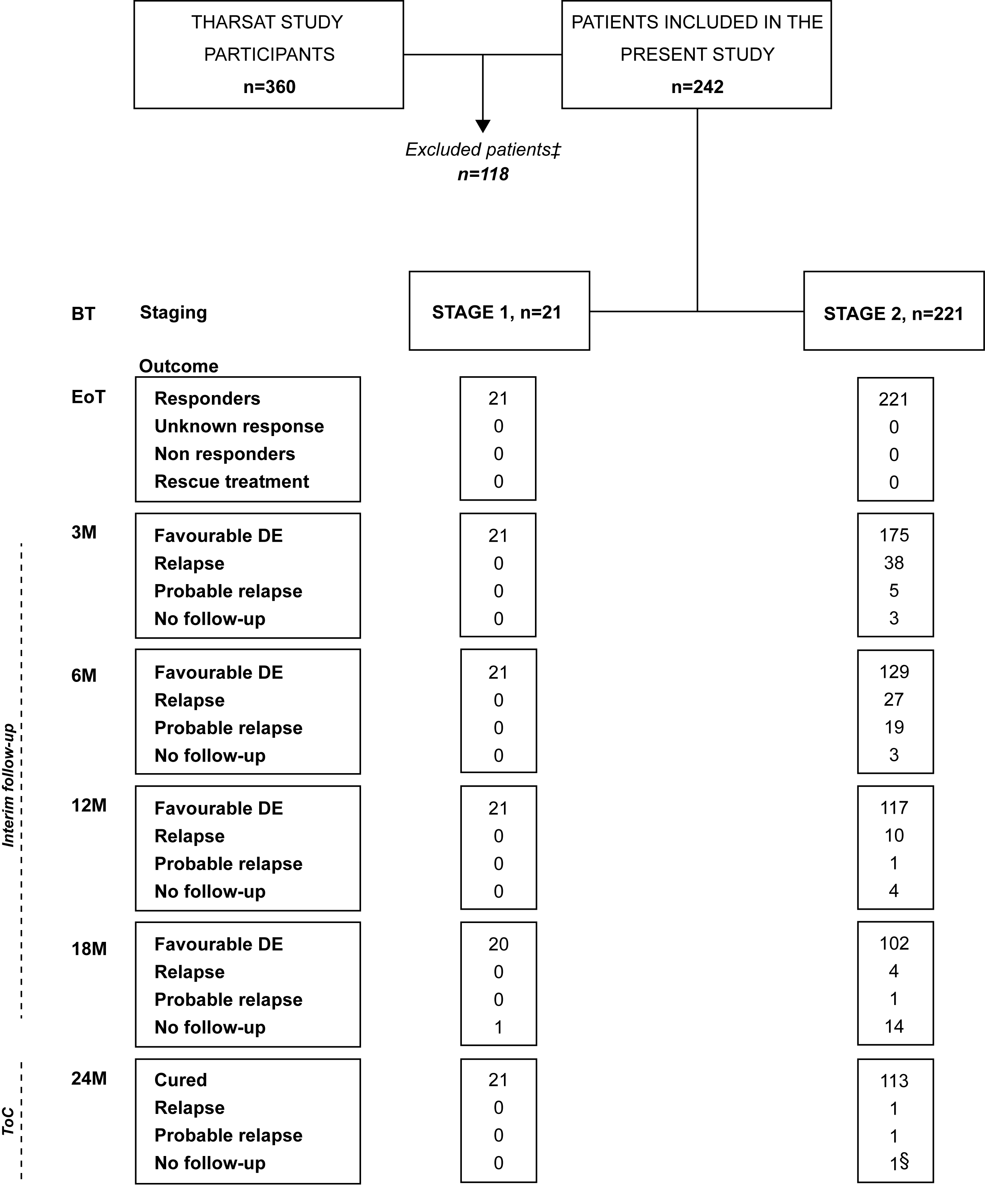

Supplement: Figure S1 — Flow-chart describing the patients investigated for the verification analysis. BT: before treatment; EoT: end of treatment; DE: disease evolution; 3 M, 6 M, 12 M, 18 M, 24 M: 3, 6, 12, 18, 24 months after treatment; ToC: test-of-cure. * Patients were included according to the criteria described by Mumba Ngoyi et al. [8]. ‡ Patients excluded from the present study: n = 8, presence of parasites in blood at the time of relapse; n = 4, stage 1 relapse; n = 37, death during the follow-up; n = 46, lost during the follow-up or missing more than one time of interim follow-up; n = 23, insufficient CSF sample. § One patient was considered cured at the end of the follow-up even if the last visit was done 18 months after treatment. Nomenclature assigned according to WHO/CDS/NTD/IDM/2007.1 [10]. (TIF) [file pntd.0002088.s001.tif]

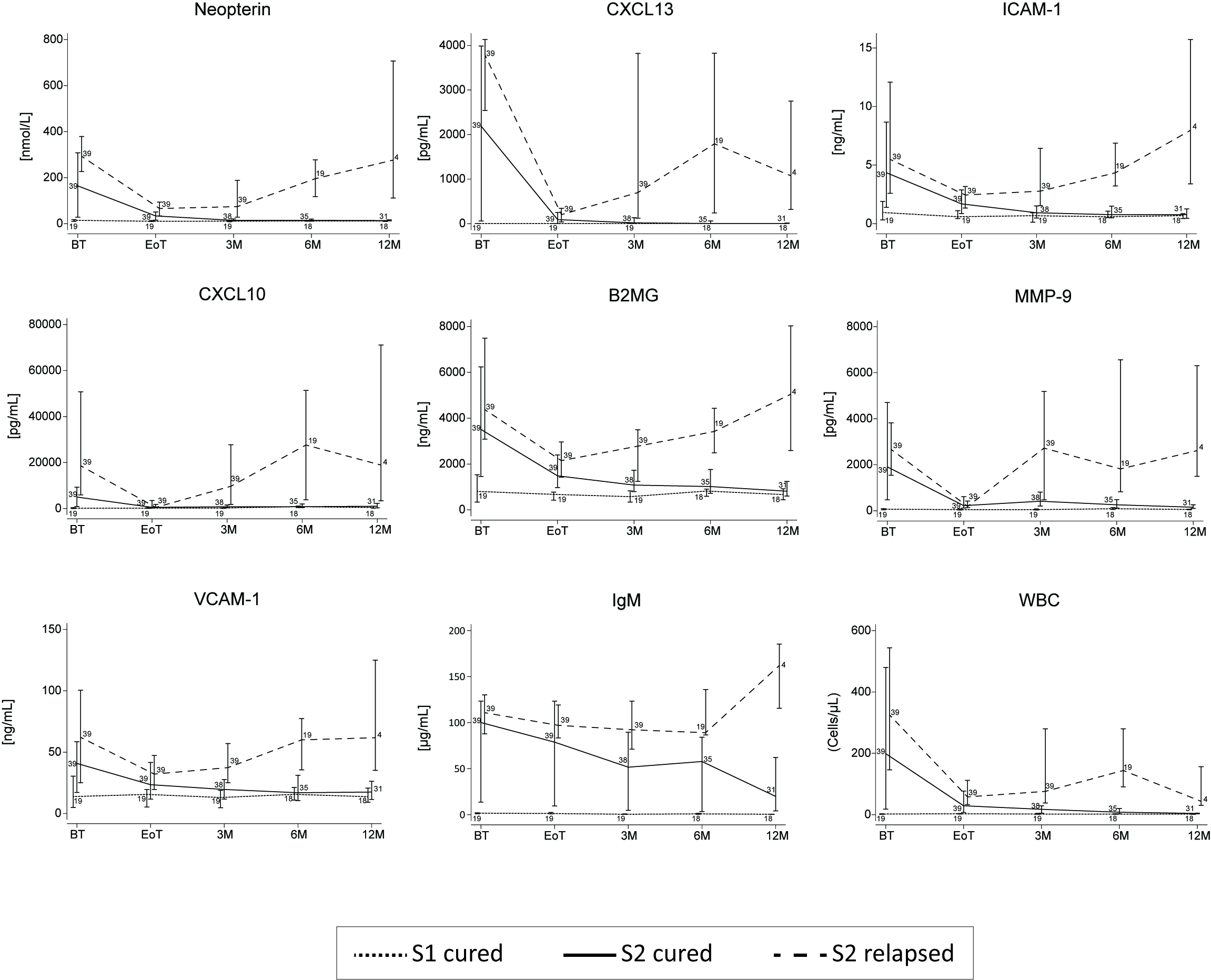

Supplement: Figure S2 — Kinetics of the eight molecules and of WBC assessed on the screening cohort. The variation in concentrations of neopterin, CXCL13, CXCL10, ICAM-1, VCAM-1, B2MG, MMP-9, VCAM-1 and IgM, as well as the number of WBC in the CSF of S1 cured patients, S2 cured patients and S2 relapsing patients are represented. Median concentrations at each time point are reported. Bars represent inter-quartile intervals. Numbers on the graphs represent the number of CSF samples assessed at each time point for each category of HAT patients. BT: before treatment; EoT: end of treatment; 3 M, 6 M, 12 M: 3, 6, 12 months after treatment. FU: follow-up. (TIF) [file pntd.0002088.s002.tif]
