# Supplementary material for: Neopterin Is a Cerebrospinal Fluid Marker for Treatment Outcome Evaluation in Patients Affected by Trypanosoma brucei gambiense Sleeping Sickness
Source: PLoS Negl Trop Dis. 2013 Feb 28;7(2):e2088. doi: 10.1371/journal.pntd.0002088 (PMC3585011; doi:10.1371/journal.pntd.0002088)
Supplement: Table S1 — Characteristics at baseline of the screening cohort. (DOC) [file pntd.0002088.s003.doc]

**Supporting table S1 Characteristics at baseline of the screening cohort**

|  | **S1 cured (n=19)** | **S2 cured (n=39)** | **S2 relapsed* (n=39)** |
| --- | --- | --- | --- |
| **Demography** |  |  |  |
| Sex, F (n)† | 10 (52.6%) | 15 (38.5%) | 15 (38.5%) |
| Age, years [mean ± SD]‡ | 38.3 [± 13.8] | 35.8 [± 15.2] | 34.6 [± 12.3] |
| **Pre-treatment CSF examination** |  |  |  |
| Trypanosome positive, n | 0 | 28 | 37 |
| WBC/µL (median, range) | 2 [1-5] | 199 [2-1940] | 325 [45-2064] |
| **Neurological signs** |  |  |  |
| Absent | 12 | 10 | 5 |
| Present | 7 | 29 | 34 |
| **Treatment** |  |  |  |
| P | 19 | 0 | 0 |
| M | 0 | 39 | 39 |

***** Time of relapse: 3M n=20; 6M n=15; 12M n=4

† No significant difference, Fisher’s exact test

‡ No significant difference, Kruskal-Wallis test

Stage 1 was defined as CSF WBC/µL ≤5 and no parasites in CSF

Stage 2 was defined as CSF WBC/µL >5 and/or parasites in CSF

P: pentamidine treatment, 4 mg/Kg/day IM for 8 days

M: melarsoprol treatment, 2.2 mg/Kg/day IV for 10 days
